# Individual and contextual factors associated with the survival of patients with severe acute respiratory syndrome by COVID-19 in Brazil

**DOI:** 10.1590/1980-549720240019

**Published:** 2024-04-19

**Authors:** Carlos Martins, Fábio Nogueira da Silva, José de Jesus Dias, Maria dos Remédios Freitas Carvalho Branco, Alcione Miranda dos Santos, Bruno Luciano Carneiro Alves de Oliveira

**Affiliations:** I Universidade Federal do Maranhão, Postgraduate Program in Collective Health – São Luís (MA), Brazil.

**Keywords:** COVID-19, Survival, Hospital care, Social environment, COVID-19, Sobrevida, Assistência hospitalar, Contexto social

## Abstract

**Objective:**

To analyze the influence of individual and contextual factors of the hospital and the municipality of care on the survival of patients with Severe Acute Respiratory Syndrome due to COVID-19.

**Methods:**

Hospital cohort study with data from 159,948 adults and elderly with Severe Acute Respiratory Syndrome due to COVID-19 hospitalized from January 1 to December 31, 2022 and reported in the Influenza Epidemiological Surveillance Information System. The contextual variables were related to the structure, professionals and equipment of the hospital establishments and socioeconomic and health indicators of the municipalities. The outcome was hospital survival up to 90 days. Survival tree and Kaplan-Meier curves were used for survival analysis.

**Results:**

Hospital lethality was 30.4%. Elderly patients who underwent invasive mechanical ventilation and were hospitalized in cities with low tax collection rates had lower survival rates compared to other groups identified in the survival tree (p<0.001).

**Conclusion:**

The study indicated the interaction of contextual factors with the individual ones, and it shows that hospital and municipal characteristics increase the risk of death, highlighting the attention to the organization, operation, and performance of the hospital network.

## RESUMO

**Objetivo:**

Analisar a influência dos fatores individuais e contextuais do hospital e do município de assistência sobre a sobrevida de pacientes com Síndrome Respiratória Aguda Grave por COVID-19.

**Métodos:**

Estudo de coorte hospitalar com dados de 159.948 adultos e idosos com Síndrome Respiratória Aguda Grave por COVID-19 internados de 01 de janeiro a 31 de dezembro de 2022 e notificados no Sistema de Informação de Vigilância Epidemiológica da Influenza. As variáveis contextuais foram relacionadas à estrutura, aos profissionais e equipamentos dos estabelecimentos hospitalares e indicadores socioeconômicos e de saúde dos municípios. O desfecho foi a sobrevida hospitalar em até 90 dias. Árvore de sobrevida e curvas de Kaplan-Meier foram utilizados para analisar a sobrevida.

**Resultados:**

A letalidade hospitalar foi de 30,4%. Idosos submetidos à ventilação mecânica invasiva e internados em cidades com baixo percentual de arrecadação de impostos apresentaram menor sobrevida quando comparados aos demais grupos identificados na árvore de sobrevida (p<0,001).

**Conclusão:**

O estudo indicou a interação de fatores contextuais com os individuais, e evidencia que características hospitalares e dos municípios aumentam o risco de óbito, destacando a atenção à organização, ao funcionamento e desempenho da rede hospitalar.

## INTRODUCTION

Severe Acute Respiratory Syndrome (SARS) can be caused by various infectious agents, including the SARS-CoV-2 virus. It is a serious condition characterized by dyspnea, respiratory rate above 30 rpm, and oxygen saturation below 93%, requiring hospital admission^1^.

Individual factors such as advanced age, male gender, presence of comorbidities and need for invasive mechanical ventilation are associated with death^2-4^. Furthermore, contextual factors related to the health service or the city where patients were admitted can also influence outcomes. However, only a limited number of studies have explored this association^3,5-7^.

Contextual factors encompass the conditions and environment of access to health care, including the structure of the system, financial aspects, and characteristics of the community. As such, their assessment is carried out in an aggregated manner rather than individually^8^. At the hospital level, the availability of financial, human, and equipment resources can impact access to healthcare services^9^. On a municipal level, socioeconomic indicators such as health, education, economic growth, social inequality, investment, and tax collection can all be taken into consideration^8^.

Studies that analyzed the relationship between contextual factors and COVID-19 mortality revealed notable findings. In Mexico, in 2020, care received in public services, as opposed to private services, was associated with increased mortality^3^, while in the United States, in 2021, the association occurred with highest municipal median income^5^. In Brazil, in 2020, higher income inequality, as measured by the Gini coefficient, and fewer municipal beds were associated with elevated mortality^6,7^. However, despite the limited number of studies, previous research did not analyze the survival outcomes of these cases; rather, they focused solely on the correlation between these variables and death^7^.

Analyzing survival, rather than just focusing on death, offers the advantage of examining the time between exposure and the event, while also handling censored data. Survival tree analysis, in particular, employs a tree-like structure with precise decision rules that are parsimonious, statistically robust, and visually interpretable^10,11^. Therefore, leveraging survival analysis, especially tree-structured models, can provide insights that facilitate a more thorough examination of the factors that contribute to the increased risk of death among patients with SARS due to COVID-19.

Given that prior studies have predominantly focused on individual risk factors for death, with only a limited examination of how contextual factors influence the dynamics of COVID-19, this study aimed to investigate the association between individual and contextual factors related to both the hospital and municipality of care concerning the survival outcomes of patients hospitalized with SARS due to COVID-19.

## METHODS

A cohort study was carried out with reported cases of SARS due to COVID-19, drawing data from the Influenza Epidemiological Surveillance Information System (*Sistema de Informação de Vigilância Epidemiológica da Influenza* – SIVEP-Gripe). SIVEP-Gripe is an information system created by the Ministry of Health to document cases and deaths from SARS and COVID-19 in Brazil. COVID-19 notifications are compulsory and encompass information from both public and private hospitals. The dataset was obtained from the OpenDataSUS website (https://opendatasus.saude.gov.br/), considering the database updated as of March 30^th^, 2023.

This study included cases reported in SIVEP-Gripe and hospitalized between January 1^st^, 2022 and December 31^st^, 2022, focusing only cases reported in 2022, as vaccination efforts throughout the country had already started. This circumstance prevented the saturation of the health system during that year, allowing for the examination of contextual variables’ influence on the survival of patients with COVID-19 in situations of greater control of the epidemic.

Information on the establishment where the cases were admitted was acquired from the National Registry of Health Establishments (*Cadastro Nacional de Estabelecimentos de Saúde* – CNES), using the Microdatasus^12^ package of the R software. Subsequently, a linkage was established between CNES data and the data from SIVEP-Gripe using the same software. This linkage process involved key fields such as: establishment identification (CNES and SIVEP-Gripe), competence (month and year of establishment update, available solely in CNES), and month and year of hospitalization of the individual (SIVEP-Gripe). Following the linkage process, details regarding the establishment where each case was admitted were incorporated for every notification recorded in SIVEP-Gripe.

Municipal variables were also gathered, including indicators comprising the 2020 Sustainable Cities Development Index – Brazil, designed to assist cities in monitoring performance in accordance with the 17 Sustainable Development Goals (SDGs) of the United Nations^13^, and the Social Inequalities Index for COVID-19 2022 (IDS-COVID-19)^14^. The linkage of these data was carried out using the municipal code. The selected indicators were those likely to be associated with health conditions and inequality within municipalities, particularly in relation to the dynamics of COVID-19 in these regions.

The study included only cases aged 20 years old or older (adults and aged people), with a final classification of SARS due to COVID-19 who were admitted to a hospital. Postpartum women, pregnant women, and those who presented missing information or typing errors on the date of hospital admission, date of discharge, information on the evolution of the case (death or discharge) were excluded. Additionally, establishments with fewer than five registered beds and those lacking information about the inpatient establishment after linkage were excluded from the analysis.

### Study variables

Individual variables represent characteristics of cases hospitalized with SARS due to COVID-19 and are categorized into: sociodemographic — gender (male, female), age (20 to 39, 40 to 59, 60 to 79, ≥80 years), race/color (White, Black, Yellow, Brown, Indigenous); clinical — Admission to the Intensive Care Unit (ICU) (Yes, No), Mechanical Ventilation (Invasive, Non-Invasive, None), Multimorbidity (Yes, No), COVID-19 vaccination schedule (Not Immunized – not vaccinated or with incomplete vaccination schedule, two doses, booster dose).

The variable multimorbidity refers to the number of comorbidities reported by patients at the time of hospitalization, which included the following risk factors: Chronic Cardiovascular Disease, Chronic Hematological Disease, Chronic Liver Disease, Asthma, *Diabetes Mellitus*, Chronic Neurological Disease, Chronic Pneumopathy, Immunosuppression, Chronic Kidney Disease, and Obesity.

The contextual variables at the hospital level obtained from the CNES include: hospital management and structure – Teaching Activity (Yes, No), Type of Management (Mixed, State, Municipal), Link with SUS (Yes, No), Size of the Hospital (Small – 5 to 49 beds, Medium – 50 to 149 beds, Large – 150 or more beds) and hospital indicators: Ratio of Doctors/bed, Nurses/bed, Physiotherapists/bed, Nursing Technicians/bed, Infusion Pump/bed, Electrocardiogram Monitor/bed, Mechanical Ventilator/bed, and Defibrillator/bed. These indicators were calculated according to the study by Botega et al.^15^.

The contextual variables at the municipal level used were: Families registered in the Single Registry for social programs (%), Life expectancy at birth (years), Municipal health budget (in *reais*, *per capita*), Population served by municipal family health teams (%), GDP *per capita* (R$ *per capita*), Gini coefficient, Access to basic health care equipment (%), Public investment (R$ *per capita*), Total revenue collected (%), IDS- COVID-19. Further details regarding the analyzed indicators can be seen in Chart 1.

**Chart 1:** Hospital and municipal indicators analyzed and calculation method.

Indicators	Indicator calculation method
Hospital level	
Availability of human resources and equipment^a^	Total doctors/Total beds Total nurses/Total beds Total physiotherapists/Total beds Total number of nursing technicians/Total beds Total infusion pumps/Total beds Total electrocardiogram monitors/Total beds Total mechanical ventilator/Total beds Total defibrillators/Total beds
Municipal level	
Families registered in the Single Registry for social programs (%)^b^	Number of resident families registered in the Single Registry with *per capita* family income of up to half the minimum wage/Total number of resident families registered in the Single Registry *100
Life expectancy at birth (years) ^b^	Average number of years of life expected for a newborn, maintaining the existing mortality pattern, in a given geographic space, in the year considered
Municipal health budget (in *reais*, *per capita*)^b^	Total health expenditure/Total population of the municipality
Population served by family health teams (%)^b^	Population served by family health teams/Total population of the municipality *100
GDP *per capita* (R$ *per capita*)^b^	Municipal GDP/Municipal population
Gini coefficient^b^	Gini coefficient by municipality
Access to basic health care equipment (%)^b^	Number of households in precarious settlements more than 1 km from basic health care equipment/Number of households in precarious settlements *100
Public investment (R$ *per capita*)^b^	Public investment by municipality/Number of inhabitants
Total revenue collected (%)^b^	Value of revenue collected in the municipality/Total value of revenue in the municipality *100
IDS-COVID-19^c^	It measures social inequalities in health associated with COVID-19 from three domains: socioeconomic, sociodemographic and difficulty in accessing health services. The quintiles of social inequality in health in municipalities range from very low (quintile 1) to very high (quintile 5).

Source: ^a^(National Registry of Health Establishments (Cadastro Nacional de Estabelecimentos de Saúde, 2022);

^b^ Sustainable Cities Development Index – Brazil (2020);

^c^ Social Inequalities Index for COVID-19 (2022).

The primary outcome of interest was survival time (in days) until in-hospital death from COVID-19. For cases that resulted in death, the survival time was calculated as the duration from hospital admission to the date of death. For those who survived, the survival time was determined from the date of hospitalization until hospital discharge. Survival time was observed up to 90 days after hospitalization; Cases with survival times exceeding 90 days or discharged before 90 days were regarded as censored. Censorship was applied at 90 days, as beyond this timeframe, cases had similar probabilities of survival.

### Data analysis

Data were analyzed using R 4.2.3 software (http://www.r-project.org/). For the variables race/color (16.5%), ICU admission (8.1%), Mechanical Ventilation (12.1%), Health Professionals (9.5%), Equipment (4.8%), and Total Revenues Collected (2.7%) that presented missing values, single imputation was performed with the Fully Conditional Specification (FCS) method implemented in the R MICE^16^ package. After the imputation procedure, a descriptive analysis was carried out, which included proportions, means, standard deviations, medians, interquartile ranges, as well as minimum and maximum values for the variables under examination.

Survival analysis was performed to evaluate factors associated with mortality from COVID-19 within 90 days of hospitalization. For this, survival trees were constructed, a non-parametric technique that incorporates tree-structured regression models to analyze survival time^17^. This technique offers flexibility by not requiring the specification of variable distributions and automatically identifying how interactions among two or more explanatory variables influence the outcome of interest^17^. Interaction is the impact of an explanatory variable on other explanatory variables and is represented by the subdivisions of the tree nodes. Furthermore, unlike linear regression models, no assumptions need to be made about the independence of explanatory variables (collinearity). If two explanatory variables are correlated, the decision tree selects the variable that provides the best split for a given node, in this case, based on a measure of node deviation between a saturated log-likelihood model and a maximized log-likelihood^18^. The terminal nodes, which represent risk groups identified by the tree, present survival curves estimated using the Kaplan-Meier method. The trees were implemented via Survival, LTRCtrees, and Party.kit packages^19-21^.

First, a tree was generated solely based on individual variables (gender, age, race/color, ICU admission, mechanical ventilation, and multimorbidities) to estimate the proportional risk for each patient. Next, three groups were established according to the tertiles of proportional risk estimated by the tree: low, moderate, and high.

After creating the tree with the individual variables, a subsequent tree was generated, incorporating the risk identified from the individual variables along with the variables related to establishments and municipalities. The objective was to discern the influence of hospital and municipal structures on the survival time of individuals.

The survival curves of cases within each terminal node were compared using the Kaplan-Meier method, along with the logrank test to ascertain differences between groups, with a significance level of 5%.

The study was approved by the Research Ethics Committee of the University Hospital of Universidade Federal do Maranhão and by the National Research Ethics Committee of the National Health Council (*Conselho Nacional de Saúde* – CNS), CAAE No. 32206620.0.0000.5086, on June 19th, 2020, as per resolutions 466/12 and 510/16 of CNS^22,23^.

## RESULTS

**Figure 1:**
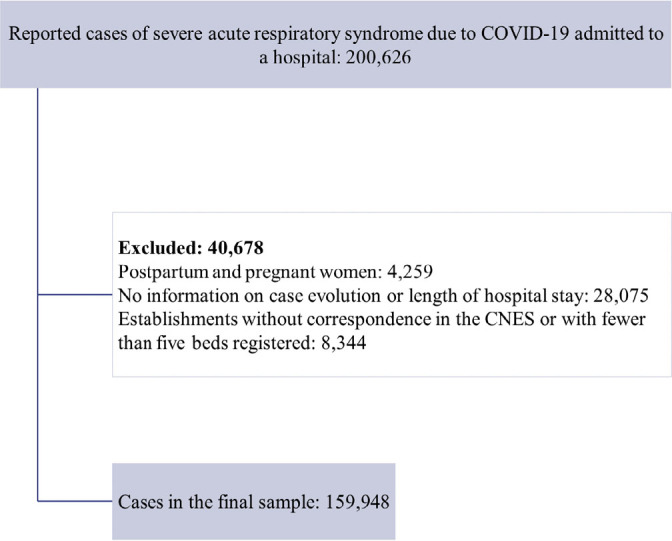
Flowchart of the sample selected for the research, Brazil, 2022.

Out of the 200,626 reported SARS cases that met the inclusion criteria, 40,678 (20.3%) were excluded, resulting in a final sample of 159,948 cases (Figure 1). Among these, 30.4% (n = 48,688) resulted in death. The median hospital stay was 6 days among censored cases and 8 days among those who died (Table 1).

**Table 1:** Sociodemographic characteristics of adults and elderly people hospitalized with SARS due to COVID-19 in Brazil in 2022.

Characteristics	Total (n=159,948) f (%)	Censored* (n=111,260) f (%)	Deaths (n=48,688) f (%)	p-value^†^
Gender					
Male	81,689 (51.1)	55,502 (67.9)	26,187 (32.1)	<0.001
Female	78,259 (48.9)	55,758 (71.2)	22,501 (28.8)	
Age (years)					
20 to 39	12,757 (8.0)	11,202 (87.8)	1,555 (12.2)	<0.001
40 to 59	28,590 (17.9)	22,604 (79.1)	5,986 (20.9)	
60 to 79	65,029 (40.7)	44,773 (68.9)	20,256 (31.1)	
80 or more	53,572 (33.5)	32,681 (61.0)	20,891 (39.0)	
Race/Color					
White	94,163 (58.9)	66,170 (70.3)	27,993 (29.7)	<0.001
Black	7,432 (4.6)	4,933 (66.4)	2,499 (33.6)	
Yellow	1,930 (1.2)	1,354 (70.2)	576 (29.8)	
Brown	56,203 (35.1)	38,658 (68.8)	17,545 (31.2)	
Indigenous	220 (0.1)	145 (65.9)	75 (34.1)	
Macro region					
Southeast	84,188 (52.6)	58,588 (69.6)	25,600 (30.4)	<0.001
South	33,479 (20.9)	23,922 (71.5)	9,557 (28.5)	
Central West	14,950 (9.3)	10,981 (73.5)	3,969 (26.5)	
North	6,019 (3.8)	4,039 (67.1)	1,980 (32.9)	
Northeast	21,312 (13.3)	13,730 (64.4)	7,582 (35.6)	
Multimorbidity					
No	113,404 (70.9)	81,672 (72.0)	31,732 (28.0)	<0.001
Yes	46,544 (29.1)	29,588 (63.6)	16,956 (36.4)	
ICU hospitalization^‡^					
Yes	58,375 (36.5)	29,481 (50.5)	28,894 (49.5)	<0.001
No	101,573 (63.5)	81,779 (80.5)	19,794 (19.5)	
Mechanical ventilation					
None	48,968 (30.6)	44,162 (90.2)	4,806 (9.8)	<0.001
Non-invasive	84,904 (53.1)	61,235 (72.1)	23,669 (27.9)	
Invasive	26,076 (16.3)	5,863 (22.5)	20,213 (77.5)	
Vaccination schedule against COVID-19					
Not immunized	45,639 (28.5)	31,078 (68.1)	14,561 (31.9)	<0.001
Two doses	54,096 (33.8)	36,413 (67.3)	17,683 (32.7)	
Booster dose	60,213 (37.6)	43,769 (72.7)	16,444 (27.3)	
Individual risk					
Low	53,346 (33.4)	48,079 (90.1)	5,267 (9.9)	<0.001
Moderate	80,526 (50.3)	57,318 (71.2)	23,208 (28.8)	
High	26,076 (16.3)	5,863 (22.5)	20,213 (77.5)	
Length of hospital stay (days)					
Mean (SD^§^)	11.12 (12.93)	10.42 (12.51)	12.74 (13.69)	<0.001
Median (Q1-Q3^//^)	7.00 (4.00-13.00)	6.00 (3.00-12.00)	8.00 (4.00-17.00)	
Minimum-Maximum	1.00-90.00	1.00-90.00	1.00-90.00	

*Hospital discharge or hospitalization for more than 90 days;

^†^Pearson’s chi-square test, for qualitative variables; and Mann-Whitney test, for quantitative variables;

^‡^Intensive care unit;

^§^Standard deviation;

^//^First and third quartile.

A higher 90-day lethality rate was observed among men (32.1%), individuals aged 80 years old or older (39%), those of black color/race (35.6%), from the Northeast region (35.6%), with multimorbidities (36.4%), who were admitted to the ICU (50.1%), and individuals who required invasive mechanical ventilation (77.9%) (Table 1).

Death was more prevalent among cases admitted to small hospitals (31.2%), those linked to SUS (32.7%), establishments without teaching activities (34.2%), and municipalities with IDS COVID of 2 to 5 (33.2%). The average ratio of defibrillators/beds in hospitals and the Gini coefficient in municipalities of individuals who died were 0.09 (±0.0) and 0.54 (±0.06), respectively, while the average proportion of collection was 24.83% (±12.44%) (Table 2).

**Table 2:** Characteristics of hospitals and municipalities where patients were hospitalized with SARS due to COVID-19 in Brazil in 2022.

Characteristics	Total f (%)	Censored f (%)	Deaths n (%)	p-value*
Size of the hospital				
Small	79,378 (49.6)	54,624 (68.8)	24,754 (31.2)	<0.001
Medium	56,356 (35.2)	39,430 (70.0)	16,926 (30.0)	
Large	24,214 (15.1)	17,206 (71.1)	7,008 (28.9)	
Linkage with SUS^†^				
No	32,414 (20.3)	25,493 (78.6)	6,921 (21.4)	<0.001
Yes	127,534 (79.7)	85,767 (67.3)	41,767 (32.7)	
Teaching activity				
No	41,901 (26.2)	27,577 (65.8)	14,324 (34.2)	<0.001
Yes	118,047 (73.8)	83,683 (70.9)	34,364 (29.1)	
Type of management				
Double	12,031 (7.5)	8,248 (68.6)	3,783 (31.4)	<0.001
State	39,700 (24.8)	27,233 (68.6)	12,467 (31.4)	
Municipal	108,217 (67.7)	75,779 (70.0)	32,438 (30.0)	
Defibrillator/bed ratio				
Mean (SD^‡^)	0.09 (0.08)	0.10 (0.08)	0.09 (0.08)	<0.001
Median (Q1-Q3^§^)	0.08 (0.05–0.12)	0.08 (0.05–0.12)	0.07 (0.05–0.11)	
Minimum-Maximum	0.00–4.00	0.00–3.40	0.00–4.00	
Gini coefficient				
Mean (SD)	0.54 (0.06)	0,54 (0,06)	0,54 (0,06)	<0.001
Median (Q1-Q3)	0.54 (0.50–0.61)	0,54 (0,50–0,61)	0,54 (0,50–0,60)	
Minimum-Maximum	0.32–0.80	0,32–0,80	0,33–0,72	
Revenue collected^//^(%)				
Mean (SD)	25.67 (12.84)	26.04 (13.00)	24.83 (12.44)	<0.001
Median (Q1-Q3)	23.54 (15.98–33.00)	24.19 (16.29–33.00)	22.26 (15.35–32.73)	
Minimum-Maximum	0.51-51.46	0.51-51.46	0.54-51.46	
IDS-COVID^¶^ (%)				
One	102,926 (64.3)	73,196 (71.1)	29,730 (28.9)	<0.001
Two to five	57,022 (35.7)	38,064 (66.8)	1?s8,958 (33.2)	

Only variables that showed interaction in the survival tree were presented in tables.

*Pearson’s chi-square test, for qualitative variables; and Mann-Whitney test, for quantitative variables;

^†^Unified Health System;

^‡^Standard deviation;

^§^First and third quartile;

^//^Percentage of revenue collected in the municipality in *reais* (R$);

^¶^Social Inequalities Index for COVID-19.

**Figure 2:**
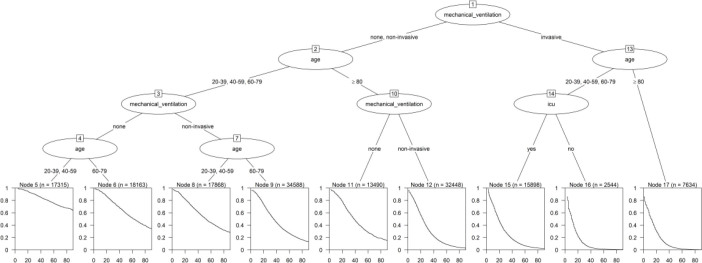
Survival tree with individual factors for death events in adults and aged people hospitalized due to COVID-19, Brazil, 2022.

Notes: The tree contains 17 nodes, 6 decision-making nodes represented by circles and 9 terminal nodes represented by squares, which contain the survival curve estimated by the Kaplan-Meier Method. Above each terminal node, the number of cases in that node (n) is specified, while at decision-making nodes the node number is identified.

The initial survival tree created from individual variables generated nine terminal nodes and employed mechanical ventilation, ICU admission, and age as decision variables (Figure 2). The stratification of groups into a categorical variable called “individual risk” occurred as follows: in the first tertile (low risk), cases belonging to nodes 5, 6, 8, and 11 and those not subjected to mechanical ventilation or adults who received non-invasive ventilation were included; the second tertile (moderate risk) comprised cases belonging to nodes 9 and 12, which included aged individuals undergoing non-invasive mechanical ventilation; Finally, the third tertile (high risk) encompassed cases undergoing invasive ventilation: nodes 15, 16, and 17. The higher the risk, the lower the survival rate of these patients.

**Figure 3:**
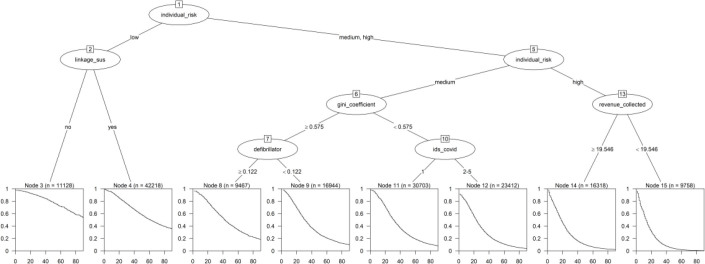
Survival tree with contextual factors for death events in adults and elderly people hospitalized due to COVID-19, Brazil, 2022.

The survival tree generated with the identified risk based on individual characteristics, hospital, and municipal characteristics included the following variables: link with SUS, defibrillator/bed ratio, Gini coefficient, revenue collected, IDS COVID, and individual risk (Figure 3). The root node, which conducts the initial division, utilized individual risk as a decision variable, ultimately identifying 8 (eight) terminal nodes.

**Figure 4:**
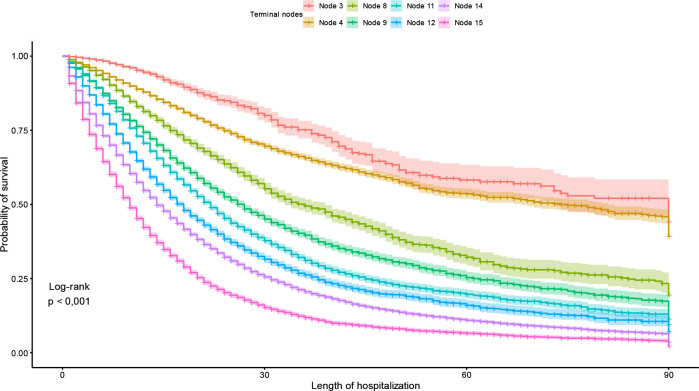
Kaplan-Meier survival curve of terminal nodes identified by the survival tree, Brazil, 2022.

Notes: Each curve represents the cases contained in the terminal nodes classified as follows: Node 3 – Mild Risk, No Link to SUS; Node 4 – Mild Risk, Link with SUS; Node 8 – Medium Risk, GINI Coefficient ≥0.575, Defibrillator/Bed Ratio ≥0.123; Node 9 – Medium Risk, GINI Coefficient ≥0.575, Defibrillator/Bed Ratio <0.123; Node 11 – Medium Risk, GINI Coefficient <0.575, IDS COVID = 1; Node 12 – Medium Risk, GINI Coefficient <0.575, IDS COVID = 2 to 5; Node 14 – High Risk, Revenues Raised ≥19.5%; Node 15 – High Risk, Revenues Raised <19.5%.

Cases classified as having mild individual risk and admitted to hospitals not linked to SUS (node 3) had a lower risk of death with a median survival time of 90 days. Cases with high individual risk who lived in cities with revenue collected less than 19.5% (node 17) had a higher risk of death and a median survival time of 10 days. The 90-day hospital survival curve showed a statistical difference between the cases of terminal nodes generated by the tree (p<0.001) (Figure 4).

## DISCUSSION

The findings suggest that individual factors, as well as factors related to the hospital structure and municipalities of care, significantly influenced the survival of patients hospitalized with SARS due to COVID-19. Their interaction defined unequal risks of death, with heightened risk observed among those who required mechanical ventilation, the aged, those admitted to hospitals linked to SUS, with limited availability of defibrillators, residing in municipalities characterized by lower Gini coefficients and percentages of revenue collected, and higher IDS-COVID.

Based on individual variables, the use of invasive mechanical ventilation and advanced age were identified as factors that reduce survival in the study group, as documented in the literature^2-4^.

Those admitted to SUS hospitals had lower survival rates, possibly due to lower availability of equipment. Before the pandemic, 72% of regions already had less than 10 ICU beds per 100,000 inhabitants, which represents limited bed availability for 61% of the Brazilian population without health insurance^24^. Despite the SUS receiving the largest number of people with conditions that require hospital admission, the system only has 48% of ICU beds in Brazil^25^.

In 2020, only 0.2% of locations lacked defibrillators, yet many regions possess up to 5 pieces of equipment per 10,000 inhabitants^26^, which may compromise patient care. Furthermore, those admitted to hospitals with a defibrillator ratio lower than 0.123 are in the North region of the country, historically characterized by limited equipment availability^24^.

SUS users often face worse socioeconomic conditions, which hinders access to health services and contribute to worse assessment of their health status^27^. Furthermore, individuals without private health insurance have a higher prevalence of chronic non-communicable diseases, which may increase the risk of developing a severe form of COVID-19^28^.

Cases hospitalized in municipalities with a Gini lower than 0.575 had lower survival rates. This coefficient is part of SDG 10 (Reduction of Inequalities) and measures the concentration of income in each municipality^29^. Although a previous study has indicated that income inequality correlated with a higher risk of death from COVID-19^30^, our findings suggest that despite these patients living in places with lower income concentration, they were admitted to hospitals with fewer health professionals and equipment, situated in municipalities with a substantial percentage of individuals with low income. Therefore, even amid lower income inequality, the lack of hospital and social resources may reduce survival. These inequalities lead to groups of people with reduced access to diagnostic tests and an elevated risk of infection, hospitalization, and death^31^.

Similar patterns were observed among individuals in municipalities that failed to meet the target and are below the green threshold of 19.7% of total revenue collected. This indicator is part of SDG 17 (Partnerships and Means of Implementation) and reflects the municipality’s capacity for tax collection, indicating its reliance on resources from the State or the Union^32^. The revenue collected by municipalities directly affects people’s health. A study delineating the evolution of municipal financing of SUS, from 2004 to 2019, shows an increase in non-own health expenses following the 2015 crisis, indicating greater fiscal dependence for healthcare funding. This means that municipalities, especially smaller ones, have become increasingly reliant on state health funds^33^. This situation becomes more challenging due to insufficient resources to cover healthcare expenses^33^.

The COVID-19 pandemic exacerbated challenges related to healthcare spending. A previous study indicated that the majority of states in the Southeast region of Brazil were not prepared for a drop in revenue, as they were already operating at the brink of their fiscal health. Indeed, in April 2020, the peak period of the pandemic, there was an impact on revenue among the states analyzed^34^. This resulted in a greater need to transfer resources from the Union to states and municipalities. However, by the end of June 2020, only 39.5 and 33.9% of the planned resources had been transferred to states and municipalities, respectively. It was not until July onwards, with already 100 thousand deaths resulting from COVID-19, that resources were transferred in greater volume^35^. Therefore, there existed a disparity between local needs and the Union’s transfer, and the delay in resource allocation underscores the Union’s lack of preparedness during a health system crisis.

IDS-COVID is another indicator that highlights social inequalities in health related to COVID-19^14^ and has been demonstrated to be a predictor of the risk of death from the disease. Cases originating from municipalities with an IDS-COVID greater than or equal to 2, that is, greater inequality, have a lower survival rate. Thus, these findings underscore the significance of identifying a cutoff point that allows greater attention to municipalities with this characteristic.

The limitations of this study are associated with the use of a secondary database, which, may potentially contain typing errors and incomplete information. Additionally, since the study focuses on hospitalized patients, the results cannot be extrapolated to all cases of COVID-19 but rather to those with a severe form of the disease. Nevertheless, inconsistent data exclusion criteria and missing data imputation techniques were employed for this analysis. Given that it is the largest national database containing information about COVID-19, it enables inference regarding the disease’s course in the Brazilian population.

Another limitation is due to the data obtained from CNES, in which 5% of the cases were hospitalized in hospitals with fewer than five beds or without correspondence with the SIVEP-Gripe data, which made their exclusion from the study necessary. The remaining variables with missing data went through the imputation process. Despite these limitations, this is one of the first studies that uses data referring to health establishments, as well as social indicators with the intention of verifying survival in this group.

This study underscores the development of models based on survival trees, enabling the integration of hierarchical structures. The algorithm employed for tree construction automatically discerns these structures, eliminating the necessity to specify the hierarchical levels of each variable within the model. Moreover, it facilitates a transparent visualization of the relationships among variables and the hierarchical arrangement of variables constituting the final model.

In conclusion, this study highlights the interaction between individual and contextual factors, revealing that hospital and municipal characteristics heighten the risk of death, even within a context of widespread vaccination that resulted in fewer hospitalized cases. These findings, when viewed through the lens of hospital and municipal indicators, underscore the ongoing challenges surrounding SUS financing and the subsequent availability of equipment and professionals. This challenge is exacerbated in municipalities characterized by a lower percentage of revenue collected and historical inequalities. Consequently, these combined factors may contribute to heightened vulnerability among patients. Hence, there is a pressing need for increased attention to the organization, functioning, and performance of the small hospital network, which often receives fewer resources. Additionally, municipalities with pronounced inequality in COVID-19-related indicators and limited resources warrant heightened scrutiny to address social and health-related demands effectively.
